# 

**Published:** 1917-10-13

**Authors:** 


### CONTENTS.

| | PAGE |
|---|---|
| **Raising a Ghost it Were Better to Lay** ... | 21 |
| **King George's Fund for Sailors** ... ... ... | 22 |
| **Hospital and Institutional News** ... ... ... | 23 |

A Chair of Tuberculosis—Spanish Officers on Hospital Ships—The Queen-Mother and Salop Infirmary—A Remodelled Cottage Hospital—Ilkeston Hospital—Cardiff Central Market Knitting Guild—Nottingham General Hospital—What to Write in a Visitors' Book—A Rent from Military Patients?—The Organisation of Employers' Subscriptions—Shell-shocked Soldiers in Plymouth—A Farmers' Hospital Fund—Cunard Steamship Company and the Wounded—A Successful Matinée Committee—The Hull Hospital Sunday Fund—Railwaymen Demand to Control a Hospital—Buxton and the Canadian Soldiers—London's Dug-outs—Legacies and Liabilities—What's in a Name?—The Manor House Hospital—The Papworth Hall Sanatorium—Shooting Lions from a Hospital's Grounds—A Novelty in Editorials—Some Beams from *The Searchlight*—The Lighter Side of Seriousness—Mrs. Atkins Writes her Letter—This Week's Drug Market.

| | |
|---|---|
| **Health and Disease** ... ... ... ... ... | 29 |

An Interpretation of Medicine to the Lay Public.

| | PAGE |
|---|---|
| **Legal Notes for Institutional Workers** ... | 30 |
| **A Hospital Day in Salonika...** ... ... ... | 31 |
| **Midwifery Teaching in Dublin** ... ... ... | 31 |

The Rotunda and Coombe Hospitals.

| | |
|---|---|
| **The Lighting of Operating Theatres** ... ... | 33 |

By Day and by Night.

| | |
|---|---|
| **What One Secretary is Thinking** ... ... | 33 |
| **Nursing Affairs in Ireland** ... ... ... ... | 34 |

The County Infirmaries and the College.

| | |
|---|---|
| **The Matrons' and Sisters' Department** ... | 35 |

Matrons in Council: X. Personality and Progress—XI. Standardise Teaching and Improve Pay—XII. The Responsibility of Matron and of Nurse.

Round the Hospitals.

The College of Nursing Moves—Anniversary of Nurse Cavell's Death—A New Form of Matron's Testimonial—Some Military Matrons' Experiences.

| | |
|---|---|
| **Food in Wartime** ... ... ... ... ... ... | 38 |
| **The Editor's Letter-Box** ... ... ... ... | 39 |
| **Hospital and Institutional Results** ... ... | 39 |
| **Matrons' and Sisters' Appointments** ... ... | 40 |
| **The Institutional Worker** ... ... (Suppl.) | 1 |

Smoking in Hospital.

